# Selective Inhibition of Human AKR1B10 by *n*-Humulone, Adhumulone and Cohumulone Isolated from *Humulus lupulus* Extract

**DOI:** 10.3390/molecules23113041

**Published:** 2018-11-21

**Authors:** Jan Moritz Seliger, Serhat Sezai Cicek, Lydia T. Witt, Hans-Jörg Martin, Edmund Maser, Jan Hintzpeter

**Affiliations:** 1 Institute of Toxicology and Pharmacology for Natural Scientists, University Medical School Schleswig-Holstein, Campus Kiel, Brunswikerstr. 10, D-24105 Kiel, Germany; martin@toxi.uni-kiel.de (H.-J.M.); maser@toxi.uni-kiel.de (E.M.); hintzpeter@toxi.uni-kiel.de (J.H.); 2 Department of Pharmaceutical Biology, Faculty of Mathematics and Natural Sciences, Christian-Albrechts-Universität zu Kiel, Gutenbergstraße 76, D-24118 Kiel, Germany; scicek@pharmazie.uni-kiel.de; 3 Department of Pharmaceutical Chemistry, Faculty of Mathematics and Natural Sciences, Christian-Albrechts-Universität zu Kiel, Gutenbergstraße 76, D-24118 Kiel, Germany; lwitt@pharmazie.uni-kiel.de

**Keywords:** aldo-keto reductases, cancer, tight-binding inhibition, selective inhibition, humulone, farnesal reduction, hops, humulus lupulus, alpha-acids

## Abstract

Hop-derived compounds have been subjected to numerous biomedical studies investigating their impact on a wide range of pathologies. Isomerised bitter acids (isoadhumulone, isocohumulone and isohumulone) from hops, used in the brewing process of beer, are known to inhibit members of the aldo-keto-reductase superfamily. Aldo-keto-reductase 1B10 (AKR1B10) is upregulated in various types of cancer and has been reported to promote carcinogenesis. Inhibition of AKR1B10 appears to be an attractive means to specifically treat RAS-dependent malignancies. However, the closely related reductases AKR1A1 and AKR1B1, which fulfil important roles in the detoxification of endogenous and xenobiotic carbonyl compounds oftentimes crossreact with inhibitors designed to target AKR1B10. Accordingly, there is an ongoing search for selective AKR1B10 inhibitors that do not interact with endogeneous AKR1A1 and AKR1B1-driven detoxification systems. In this study, unisomerised α-acids (adhumulone, cohumulone and *n*-humulone) were separated and tested for their inhibitory potential on AKR1A1, AKR1B1 and AKR1B10. Also AKR1B10-mediated farnesal reduction was effectively inhibited by α-acid congeners with K_i_-values ranging from 16.79 ± 1.33 µM (adhumulone) to 3.94 ± 0.33 µM (*n*-humulone). Overall, α-acids showed a strong inhibition with selectivity (115–137 fold) for AKR1B10. The results presented herein characterise hop-derived α-acids as a promising basis for the development of novel and selective AKR1B10-inhibitors.

## 1. Introduction

In (phyto-)pharmacology and nutritional medicine, beer and its constituents have been subject to numerous epidemiological and molecular studies, not least in order to evaluate the manifold effects of its main flavouring ingredient, the female inflorescences of the hop plant (*Humulus lupulus*). In particular, hop-derived chalcones (xanthohumol), prenylflavonoids (isoxanthohumol, 8-prenylnaringenin) and (iso-) α- and β-bitter acids ((iso-)humulone and (iso-)lupulone) are among the bioactive compounds accounting for various modes of action in the prevention or potential treatment of many (lifestyle) diseases. These include metabolic and inflammatory diseases as well as carcinogenesis [1,2,3,4,5]. 

**Figure 1:**
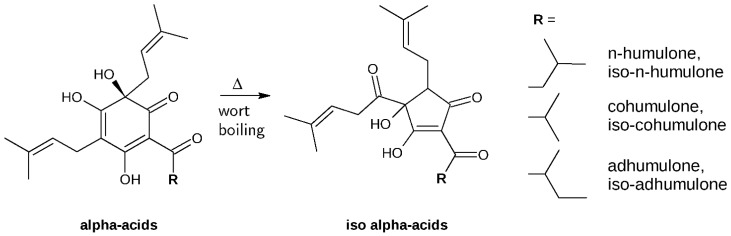
Structures of α-acids (*n*-humulone = compound **3**, cohumulone = compound **2** and adhumulone = compound **1**) and iso-α-acids (isohumulone, isocohumulone, and isoadhumulone) after thermal isomerisation through wort boiling.

Iso-α acids (isoadhumulone, isocohumulone, isohumulone) derived from thermic isomerization of their precursors (adhumulone (compound **1**), cohumulone (compound **2**), *n-*humulone (compound **3**)) during wort boiling are counted among the most abundant classes of phenolics in beer with concentrations ranging from 0.6 to 100 mg/L (Figure 1) [4,6]. Unisomerized α-acids are comparatively less abundant (1.7 mg/L) [6], their concentration might, however, increase due to processes called late or dry-hopping, where hop cones or pellets are added either near the end of the boiling process or even later, at low temperatures, before the product is packaged [6]. 

The chemopreventive effect of α- and iso-α-acids on biological systems has been investigated in earlier studies and extensively reviewed by Gerhäuser [4]. Apart from their antibiotic capacities, and antiangiogenic and antidiabetic properties, α-acids have been reported to interfere with carcinogenesis. For example, in human hepatocarcinoma cells, α-acids significantly reduced phosphorylation of NfκB as well as AP-1 and ERK1/2 activity, thus reducing migration and proliferation [1]. 

Prenylation of GTP-binding proteins, a process occurring further upstream of the involved RAS-RAF-MEK-ERK (MAPK) pathway, may cause aberrant activation of RAS or RAS-like proteins, which contributes to the development of different malignancies including glioblastoma, hepatocellular carcinoma and pancreatic cancer [7,8,9]. As the mechanism of protein prenylation requires intermediates from the cholesterol metabolism, it is, among other reactions, strongly dependent on the reduction of the isoprenoids geranylgeranyl and farnesal in order to provide proper covalent bonding to the C-terminal cysteines of the target proteins [10,11]. These isoprenoids fall within the specific substrate spectrum of aldo-keto reductase member 1B10 (AKR1B10), an NADPH-dependent oxidoreductase that has been shown to play a pivotal role in the prenylation-dependent activation of KRAS and RAS-like proteins by mediating the reduction of geranylgeranyl and farnesal to their respective alcohols [9,12]. Hence, AKR1B10 has not only become a biomarker (as it is upregulated in various types of cancer), but has also turned into an attractive pharmacological target in cancer prevention and treatment. Accordingly, numerous studies have been conducted in the search for new selective inhibitors for AKR1B10 [13,14,15,16].

Apart from the aforementioned lipids and unlike other members of the aldo-keto reductase family that accept sugar and lipid aldehydes, steroid hormones, prostaglandins and xenobiotics as their substrates, AKR1B10 only reduces selected carbonyls and retinal (retinaldehyde) to their corresponding alcohols [13,17]. However, due to its capacities of detoxifying reactive carbonyl compounds (which would otherwise induce apoptosis) and carbonyl group containing chemotherapeutics, it contributes to different resistance mechanisms in cancer cells when upregulated [18,19,20,21]. By reducing retinal to retinol, AKR1B10 prevents retinal from entering the retinoic acid pathway, thereby diminishing the cellular potential to regulate differentiation and proliferation [22,23,24]. 

Two other members of the same superfamily AKR1B1 and AKR1A1 are closely related to AKR1B10, sharing around 71% and 48% of sequence identity on the protein level, respectively [25]. Furthermore, they are both involved in other detoxifying mechanisms and are necessary to maintain homeostasis of the glucose metabolism [26]. Hitherto, cross-inhibition is a major pitfall in the development of inhibitors specific to either one of these enzymes as it oftentimes accounts for severe side effects in their clinical application [14,27]. 

Herein, we report the in vitro inhibitory effects of the three most prevalent, hop-derived α-acids (compound **1**–**3**) on the catalytic activity of human AKR1B10, AKR1B1 and AKR1A1. The selective binding behaviour for AKR1B10 renders these natural-based compounds promising structural analogues of new AKR1B10 inhibitors. 

## 2. Results and Discussion

In recent research hop-derived prenylflavonoids, including the most prominent hop-compounds xanthohumol and 8-prenylnaringenin, have been subject to a variety of studies in order to elucidate the beneficial effects of these substances in certain disease models. However, experimental data on the biological interaction potential of hop bitter acids, such as (iso-)-α-acids, are relatively scarce [4]. This is especially true for non-isomerized α-acids, which are up to 10-fold (≈10 µM) enriched in many late- or dry-hopped types of beer [28]. Lately, these techniques have become more prominent in the craft beer industry, which, in the future, might lead to increased plasma levels of α-acids following the consumption of certain types of beer. 

In this study, mixtures of iso-α-acids and α-acids and the three purified α-acids, compound **1**, **2** and **3**, were evaluated as inhibitors of the three related human aldo-keto reductases AKR1A1, AKR1B1 and AKR1B10. For comparability reasons and in order to investigate selectivity, DL-glyceraldehyde served as a common test substrate (Table 1, Table 2 and Table 3). Compared to the iso-α-acid mixture, the mixture of α-acids showed superior inhibitory effects with respect to all enzymes tested (Table 1). A slight selectivity for AKR1B1 was observed with the iso-α-acid mixture (Table 1). 

However, AKR1B10 inhibition was up to 115 (ratio AKR1A1/AKR1B10)–137 times (ratio AKR1B1/AKR1B10) stronger than inhibition of AKR1A1 and AKR1B1, respectively, when a mixture of α-acids was applied (Table 3).

Shindo et al. [29] report on the inhibitory effect of iso-α-acids on AKR1B1 at lower concentrations (48% inhibition at 33 µg/mL). IC_50_ values of iso-α-acids for AKR1B1 in the present study were somewhat higher (100.30 ± 6.03 µg/mL) than reported by Shindo et al. The observed discrepancy to the present study might have been due to the source and quality of the inhibitor as well as due to different purification conditions of the recombinant enzyme. In the present study a 30% (*w/w*) prediluted standardised solution of iso-α-acids produced from CO_2_ hop extract has been used, whereas Shindo et al. used international calibration standard iso-α-acids. Moreover, the concentrations of the single iso-α-acid congeners in the mixture were not further evaluated in both studies and might have influenced the respective IC_50_ values as well. Unlike in the present study, Shindo et al. used recombinant AKR1B1 from a eukaryotic expression system, which might have affected the binding behaviour of the inhibitor through posttranslational modifications that are not present in an enzyme derived from the expression system used in this study. 

Additionally, potential synergistic effects with other substances found in the hop extract could have also affected AKR1B1 activity. In fact, hop-specific compounds such as xanthohumol, isoxanthohumol and 8-prenylnaringenin have been reported to be strong inhibitors of AKR1B1 and its related reductase AKR1B10 [30]. 

**Figure 2:**
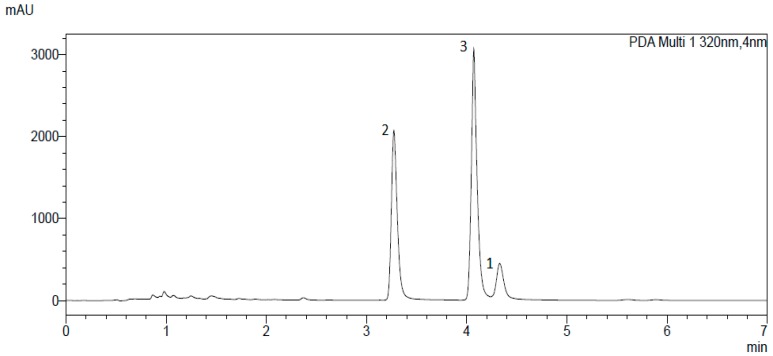
Chromatogram of α-acid separation. Compound **1** = adhumulone; compound **2** = cohumulone; compound **3** = *n*-humulone. See text for further details.

Due to the promising results of the α-acid extract, AKR-inhibition of the single compounds was of particular interest. UHPLC analysis of the extract showed three dominating peaks (Figure 2) corresponding to the major α-acids (*n*-humulone (**3**), cohumulone (**2**) and adhumulone (**1**)) found in hops [28]. Thereupon, α-acids were separated by preparative column chromatography, yielding compound **2** as well as a mixture of compounds **1** and **3** in a first step. Subsequent semi-preparative HPLC led to the isolation of the remaining two main compounds, **1** and **3**. All three substances were further analysed for their inhibitory potential.

Unlike the iso-α-mixture, IC_50_ determination of the single hop compounds isolated from the α-acid-mixture showed AKR1B10 selectivity (Table 3). Among the three compounds tested, AKR1B10 inhibition by compound **2** was the strongest (IC_50_ = 1.35 ± 0.07 µM). In case of AKR1A1 and AKR1B1, inhibition was less than 50 % at 100 and 125 µM, respectively (Table 2). Similarly, humulone from beer hop extract has been shown to also selectively inhibit the inflammatory modulator cyclooxygenase-2 (IC_50_ = 1.60 µM), whereas homologous cyclooxygenase-1 was not inhibited at concentrations below 10 µM [31]. Interestingly, arachidonic acid, the primary substrate of cyclooxygenase-1 and 2, effectively inhibits AKR1B10 at nanomolar concentrations (K_i_ = 0.26 µM) [32]. Hence, similarities in the binding behaviour of humulone and unsaturated fatty acids to these enzymes might as well pinpoint towards common inhibitory mechanisms underlying the effects observed with AKR1B10.

We further determined inhibition parameters of compounds **1**, **2** and **3** during farnesal reduction by AKR1B10, a process that has been postulated to be crucial for the prenylation and thus activation of several RAS- or RAS-like proteins during carcinogenesis [9]. Thus, inhibiting one of the key enzymes in protein prenylation constitutes an opportunity to interfere with RAS-driven carcinogenesis. Remarkably, an isovaleryl group at the C4 position of compound **3** seems to improve its inhibitory performance on AKR1B10: IC_50_ values were approximately 4-fold lower with compound **3** (7.78 ± 0.43 µM) compared to its related compounds **1** (29.27 ± 1.53 µM) and **2** (29.78 ± 1.72 µM), which show slightly different acylic moieties at the C4 position (Figure 1). Also, prenylation of the inhibitor seems to positively influence binding behaviour: a ring contraction occurring during isomerisation of α-acids leads to the introduction of a carbonyl group at the C6-prenyl residue, which might explain the comparatively lower inhibition capacities of iso-α-acids. A similar effect has been observed with γ-mangostin, another potent natural AKR1B10-inhibitor from mangosteen (*Garcinia mangostana*) (IC_50_ = 0.018 µM), and two related xanthones 1,5-dihydroxy-2-isoprenyl-3-methoxyxanthone [(1,5-DIMX) (IC_50_ > 10 µM) and 1,7-dihydroxy-2-isoprenyl-3-methoxyxanthone (1,7-DIMX) (IC_50_ = 0.85 µM)]. In this case, the loss of an isoprenyl group in 1,5-DIMX and 1,7-DIMX might have also contributed to deteriorating the inhibitory effect when compared to the parent substance [16]. 

With respect to the mechanism of inhibition, Zhang et al. suggested a more accessible anionic site at TRP112, allowing the entrance of more bulky and rigid inhibitors at the broader active site of AKR1B10 [33]. Molecular docking experiments have been performed for the three reductases in order to clarify the binding behaviour and specificity of the respective inhibitors. Even though in silico analyses indicated a strong inhibition at the active sites of the enzymes, the mechanism favouring AKR1B10 inhibition over inhibition of the other two enzymes could not ultimately be resolved (data not shown).

**Figure 3:**
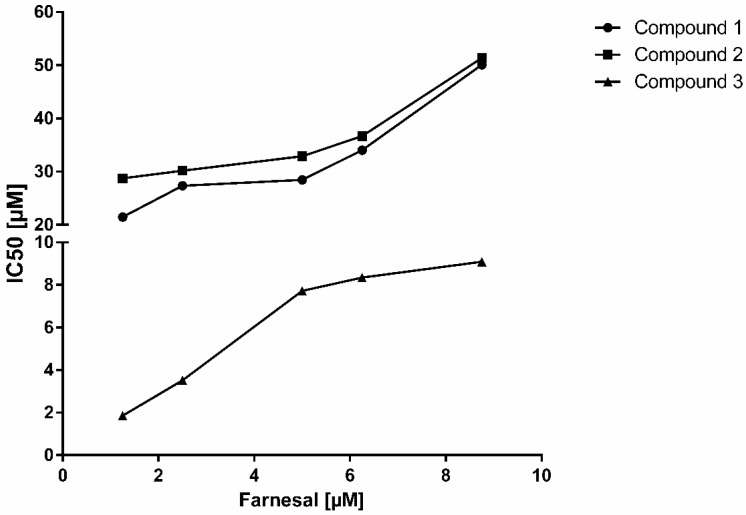
IC_50_-values of the isolated hop compounds for AKR1B10-catalysed farnesal reduction as a function of substrate concentration [compound **1** (adhumulone) (circles), compound **2** (cohumulone) (squares) and compound **3** (*n*-humulone) (triangles)].

**Figure 4:**
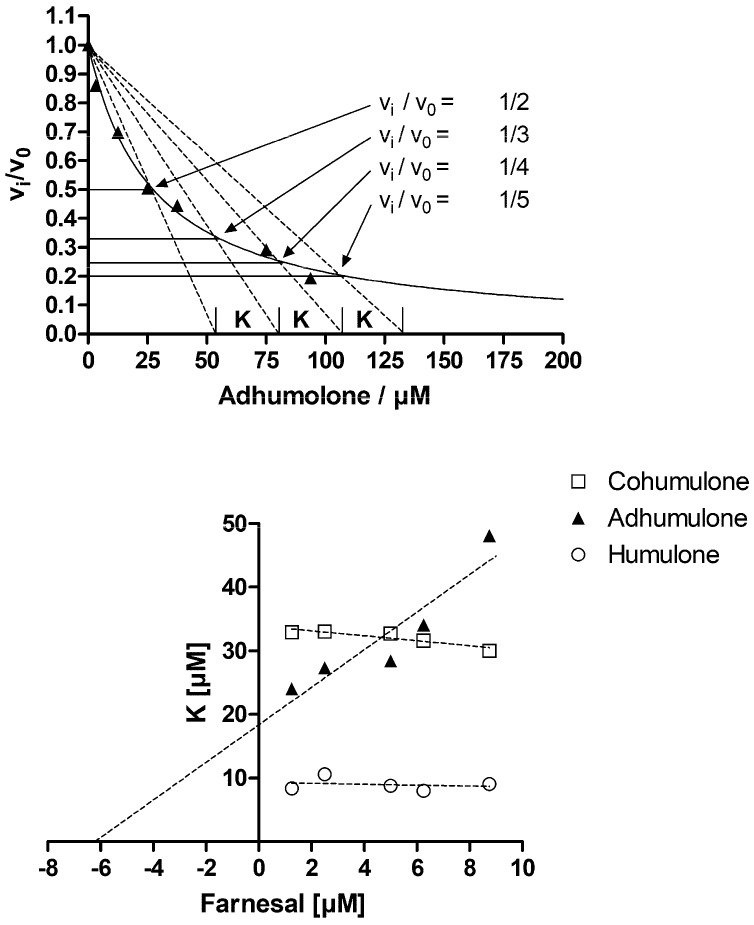
Determination of inhibition mode. To determine the mode of inhibition the normalized velocity is plotted as a function of inhibitor concentration as exemplified in the upper diagram (farnesal = 1.25 µM). The data were fitted to v_i_/v_0_ = 1/(1 + ([I]/IC_50_)) and lines are drawn from [I] = 0 to the intersection of v_i_/v_0_ = ½ and so on. The intersection of the dotted lines with the x-axis define the constant K as indicated [34,35]. This was done for all used farnesal and inhibitor concentrations and K was plotted as a function of substrate concentration (lower diagram). This secondary plot shows an increase of K with substrate concentration, which is characteristic for a competitive inhibitor (adhumulone). In the case of a non-competitive inhibitor K would be independent of substrate concentration (humulone and cohumulone).

Among the natural-based derivatives serving as selective AKR1B10 inhibitors, hop-derived α-acids have not been investigated so far. For the substances tested in this study, non-competitive and competitive modes of inhibition have been observed (Table 4, Figure 3 and Figure 4). With regard to the inhibition of AKR1B10 mediated farnesal reduction (K_M_ = 5 µM), compound **3** was the best inhibitor with a relatively low K_i_ (3.94 ± 0.33 µM) when compared to its congeners, compound **2** (K_i_ = 16.53 ± 1.74 µM) and **1** (K_i_ = 16.79 ± 1.33 µM). 

Based on the IC_50_ and K_i_ values stated herein, inhibition of AKR1B10 through α-acids during glyceraldehyde or farnesal reduction, might also be compared to the inhibitory efficiency of unsaturated fatty acids on this enzyme (K_i_-values ranging from 0.24 to 1.1 µM). Unsaturated fatty acids show a competitive inhibition pattern with a specificity towards AKR1B10 [32]. Accordingly, the mechanism of action suggested by Hara et al. [32], which involves the presence of relatively long chain of carbon‒carbon double bonds interacting with the enzyme’s active site, might in parts also apply for humulone and its three isoprenoid side chains. However, X-ray diffraction experiments would help to further clarify the actual binding mechanism.

Under physiological conditions, the inhibitory effect might be further strengthened with increasing uptake of lipophilic congeners. In this context, a study by Cattoor et al. reported efficient epithelial absorption of α-acids, which might as well point towards a rather high bioavailability [36,37]. Data on the bioavailability on α-acids in animal models are currently lacking; however, the metabolically relevant concentrations (K_i_, IC_50_) stated herein, fall within the bioavailable spectrum of iso-α-acids reported by others: a study on the bioavailability of iso-α-acids in rabbits report of cumulative iso-α-acid concentrations between 7 and 20 µM [37]. These concentrations seem sufficient for having an impact on AKR1B10-mediated farnesal reduction. In conjunction with the development of functional foods, there is increasing evidence that prenylation of a target compound raises its bioavailability [38]. As prenylation occurs in both unisomerised and isomerised α-acids, an overall higher bioavailability of these compounds might be expected. In vivo, steadily high concentration levels would be especially important when a competitive mode of inhibition is observed (Table 4, Figure 3 and Figure 4).

In general, research has made great advances in terms of designing new, effective AKR1B10 inhibitors. Unfortunately, though, the clinical safety of their use has in many cases not been evaluated yet [14]. In contrast, hops and hop-derived bitter acids are considered free for consumption and generally recognized as safe for oral intake [28,39,40]. Therefore, α-acids may yield the potential to serve as an alternative basis for the development of AKR1B10-inhibitors. 

In conclusion, the results presented in this study identify α-acids as potent inhibitors with a selectivity for AKR1B10 versus homologous AKR1A1 and AKR1B1. Of the three α-acid congeners tested, inhibition by compound **3** showed the strongest inhibition. Moreover, there is evidence of isoprenoid side chains tending to affect the binding behaviour of AKR1B10 inhibitors. With regard to AKR1B10 selectivity, our results provide a structural basis for the development of future QSAR models and new drugs/inhibitors targeting cancers characterized by AKR1B10-specific actions or AKR1B10 upregulation.

## 3. Materials and Methods

### 3.1. Chemicals and Reagents

Organic solvents for chromatography, MS grade water and MS grade formic acid were obtained from VWR (Darmstadt, Germany). Organic solvents used for preparative, semi-preparative and analytical chromatography were of gradient grade quality and water was bi-distilled water. Solvents used for LC-MS analyses were of MS grade quality. Formic acid used for chromatography was of MS grade quality. NADPH was obtained from Carl Roth GmbH & Co. KG (Karlsruhe, Germany). DL-glyceraldehyde and farnesal were purchased from Sigma-Aldrich Co. (St. Louis, MO, USA). A standardised solution of iso-α-acids produced from CO_2_ hop extract (30% *w*/*w*) was obtained from Barth Haas UK Limited (Tonbridge, UK). Mixtures of α-acids were kindly provided by Dr. Martin Biendl (Hopsteiner—HHV GmbH, Mainburg, Germany). 

### 3.2. Isolation and Identification of α-Acids

Preparative LC was accomplished using a Büchi PrepChrom C-700 and Büchi PrepChrom C18 column (15 μm, 250 × 30.0 mm) (Büchi, Germany). Semi-preparative chromatography was carried out on a Waters Alliance e2695 Separations Module equipped with an Alliance 2998 PDA detector and a WFC III fraction collector (Waters, Milford MA, USA) using a Phenomenex Aqua column (5 µm. 250 × 10.0 mm). Fractions and pure compounds were analysed by a VWR-Hitachi Chromaster Ultra RS (VWR, Darmstadt, Germany) using a Nucleodur C18 Pyramid column (5 µm, 250 × 4.6 mm). LC-MS was conducted with a Shimadzu Nexera X2 UHPLC system and a Shimadzu LC-MS 8030 triple quadrupole mass spectrometer using electrospray ionization (Shimadzu, Kyoto, Japan) and a Nucleodur C18 Gravity-SB column (1.8 µm, 100 × 2.0 mm). LC-conditions: isocratic elution with 0.4 mL/min at 30 °C using 0.1% formic acid (30%) and acetonitrile (70%). MS-conditions: nebulizer gas 3 L/min, drying gas 15 L/min, DL temperature 250 °C, heat block temperature 400 °C. Mass range was 100 to 1000 *m/z*. 

900 mg of α-acids extract were dissolved in 15 mL of 85% methanol and subjected to preparative chromatography using 0.025% formic acid in water and methanol (15:85) yielding 5 fractions. Fraction 2 contained 125 mg of cohumulone (**2**) while fraction 4 yielded 67 mg of *n*-humulone (**3**) and 28 mg of adhumulone (**1**) after separation by semi-preparative chromatography using 0.025% formic acid in water and acetonitrile (30:70). Compounds were assigned according to their molecular masses and their retention times in comparison with a reference chromatogram [41], as well as their relative amounts in the extract used for isolation.

### 3.3. Preparation of Recombinant Proteins

The carbonyl-reducing enzymes AKR1A1, AKR1B1, AKR1B10 were prepared in an *Escherichia coli* expression system according to previously published methods: plasmids of AKR1A1 and AKR1B1 were friendly gifts from Prof. Dr. Vladimir Wsol [42] and Dr. Nina Kassner; information about production and purification of AKR1B10 [19] has been published before (sequences of all obtained plasmids containing the specific inserts were verified by sequencing (MWG Eurofins)). The plasmids were then transformed in *E. coli* BL21 (DE3) cells. For overexpression of 6× His-tagged enzymes, a 400 mL culture (containing the appropriate antibiotic; plasmid dependent) was grown to optical density of 0.6 at 600 nm at 37 °C. Expression was induced by adding isopropyl-1-thio-galactopyranoside to the culture medium (final concentration of 1 mM). After 3 h, cells were harvested by centrifugation (6000× *g*, 15 min) and resuspended in 20 mL PBS-I buffer (20 mM Na_2_H_2_PO_4_, 500 mM NaCl, 10 mM imidazole, 10% *v/v* glycerol, pH 7.4). Cell disruption was performed by ultrasonication with cooling on ice to avoid heating. The sample was subsequently centrifuged at 100,000× *g* at 4 °C for 1 h. The obtained supernatants containing the respective enzymes were purified using Ni-affinity chromatography (ÄKTA-Purifier; Amersham Pharmacia, Uppsala, Sweden) using PBS-II buffer (20 mM Na_2_H_2_PO_4_, 500 mM NaCl, 500 mM imidazole, 10% *v/v* glycerol, pH 7.4). Purification progress was monitored by SDS-PAGE of the obtained fractions (not shown). Enzyme concentrations were determined using a Qubit 2.0 fluorometric quantitation system (Life Technologies, Carlsbad, CA, USA) according to the manufacturer’s instructions. 

### 3.4. Determination of Inhibition Parameters Using Test Substrates

Catalytic properties were determined by measuring the decrease in absorbance at 340 nm (Cary 100 scan photometer, Varian, CA, USA). A reaction mixture without inhibitor consisted of different concentrations of DL-glyceraldehyde or farnesal, 200 μM NADPH, 0.1 M NaH_2_PO_4_ buffer (pH 7.4) and an appropriate amount of enzyme in a total assay volume of 0.8 mL. Final enzyme concentrations in the assay ranged from 222 nM (AKR1A1) to 899 nM (AKR1B10). K_M_ values were obtained by fitting the kinetic data (mean ± SD from at least three experiments) to the Michaelis‒Menten model, as implemented in GraphPad Prism6 (GraphPad Software Inc., La Jolla, CA, USA). 

For inhibition studies, stock solutions of inhibitors were prepared in H_2_O (iso-α-acid mixture) and DMSO (α-acid mixture and compounds **1**–**3** purified from the same mixture). The final concentration of DMSO in the assay was ≤ 1% and did not affect enzyme activity. When collecting data for dose–response curves initial velocities of DL-glyceraldehyde or farnesal reduction (substrate concentration at K_M_) in the presence of inhibitors were assayed as described above. The percentage of inhibition was calculated considering the activity in the absence of inhibitor to be 100%.

Initially, the half maximal inhibitory concentrations (IC_50_ values) were determined for each inhibitor in presence of each enzyme, using the shared substrate DL-glyceraldehyde (set to their specific K_M_; 3.6 mM, 50 μM and 4 mM for AKR1A1, AKR1B1 and AKR1B10, respectively) to assess specificity amongst the structurally similar members of the AKR-superfamily.

For IC_50_ determination, experimental data were normalised and fitted to a sigmoidal curve as implemented in GraphPad Prism6 (GraphPad Software Inc., La Jolla, CA, USA). Whenever tight-binding inhibition was observed, the inhibition constant Ki was determined by fitting inhibition data to the Morrison equation [43]. In order to verify the inhibitory potency, farnesal as an enzyme-specific physiological substrate for AKR1B10 (farnesal; K_M_ = 5 µM) was used to determine inhibition parameters. Enzyme inhibition parameters were assayed as described above. The inhibition mechanism of each compound for AKR1B10 was analysed by plotting IC_50_-values at different substrate concentrations (at least five inhibitor and substrate concentrations) [43,44]. All data obtained were plotted and analysed using GraphPad Prism6 (GraphPad Software Inc., La Jolla, CA, USA).

## Figures and Tables

**Table 1:** IC_50_ values of an iso-α-acid solutions and an α-acid mixture for the respective reductases. IC_50_ values are presented as mean ± SD of at least three experiments.

Enzyme	Iso-α-Acid Solution IC_50_ [µg/mL]	α-Acid Mixture IC_50_ [µg/mL]
AKR1B10	127.90 ± 9.79	0.42 ± 0.02
AKR1B1	100.30 ± 6.03	57.47 ± 1.76
AKR1A1	163.00 ± 8.96	48.23 ± 1.81

**Table 2:** IC_50_ and K_i_ values of the isolated hop-compounds for the respective reductases. IC_50_ and K_i_ values are presented as mean ± SD of at least three experiments. (n. d. = not determined).

Enzyme	AKR1A1		AKR1B1		AKR1B10	
Substrate	Glyceraldehyde [3.6 mM]		Glyceraldehyde [50 µM]		Glyceraldehyde [4.0 mM]	
Parameter	IC_50_	K_i_ (Morrison)	IC_50_	K_i_ (Morrison)	IC_50_	K_i_ (Morrison)
Compound **1**	≥100 µM	n. d.	>125 µM	n. d.	5.41 ± 0.42 µM	3.27 ± 0.52 µM
Compound **2**	>100 µM	n. d.	>125 µM	n. d.	1.35 ± 0.07 µM	0.70 ± 0.09 µM
Compound **3**	>100 µM	n. d.	>125 µM	n. d.	1.94 ± 0.10 µM	0.98 ± 0.12 µM

**Table 3:** AKR1B10 selectivity of the α-acid-mixture and its isolated compounds expressed as IC_50_-ratios of AKR1A1/AKR1B10 and AKR1B1/AKR1B10.

Compounds	Ratio AKR1A1/AKR1B10	Ratio AKR1B1/AKR1B10
α-acid mixture	115	137
Compound **1**	≥19	>23
Compound **2**	>74	>93
Compound **3**	>52	>64

**Table 4:** IC_50_ and K_i_ values of AKR1B10 mediated farnesal reduction with isolated hop-compounds. IC_50_ and K_i_ values are presented as mean ± SD of at least three experiments.

Enzyme	AKR1B10		
Substrate	Farnesal [5 µM]		Mode of inhibition
Parameter	IC_50_	K_i_	
Compound **1**	29.27 ± 1.53 µM	16.79 ± 1.33 µM	competitive
Compound **2**	29.78 ± 1.72 µM	16.53 ± 1.74 µM	non-competitive
Compound **3**	7.78 ± 0.43 µM	3.94 ± 0.33 µM	non-competitive

## Abbreviations

1,5-DIMX	1,5-dihydroxy-2-isoprenyl-3-methoxyxanthone
1,7-DIMX	1,7-dihydroxy-2-isoprenyl-3-methoxyxanthone
AKR	Aldo-keto reductase
AP-1	Activator protein 1
DMSO	Dimethyl sulfoxide
ERK-1/2	Extracellular signal-regulated kinase 1/2
GTP	Guanosine triphosphate
HPLC	High-performance liquid chromatography
KRAS	KRAS proto-oncogene
LC	Liquid chromatography
LC-MS	Liquid chromatography-mass spectrometry
MAPK	Mitogen-activated protein kinase
MEK	Mitogen-activated protein kinase
NADPH	Nicotinamide adenine dinucleotide phosphate
NFκB	Nuclear factor ‘kappa-light-chain-enhancer’ of activated B-cells
QSAR	Quantitative structure-activity relationship
RAF	Rapidly accelerated fibrosarcoma
RAS	Rat sarcoma
UHPLC	Ultra-high-performance liquid chromatography

## References

[1] Saugspier M., Dorn C., Czech B., Gehrig M., Heilmann J., Hellerbrand C. (2012). Hop bitter acids inhibit tumorigenicity of hepatocellular carcinoma cells in vitro. Oncol. Rep..

[2] Ano Y., Dohata A., Taniguchi Y., Hoshi A., Uchida K., Takashima A., Nakayama H. (2017). Iso-α-acids, Bitter Components of Beer, Prevent Inflammation and Cognitive Decline Induced in a Mouse Model of Alzheimer’s Disease. J. Biol. Chem..

[3] Shimamura M., Hazato T., Ashino H., Yamamoto Y., Iwasaki E., Tobe H., Yamamoto K., Yamamoto S. (2001). Inhibition of Angiogenesis by Humulone, a Bitter Acid from Beer Hop. Biochem. Biophys. Res. Commun..

[4] Gerhäuser C. (2005). Beer constituents as potential cancer chemopreventive agents. Eur. J. Cancer.

[5] Van Cleemput M., Heyerick A., Libert C., Swerts K., Philippé J., De Keukeleire D., Haegeman G., De Bosscher K. (2009). Hop bitter acids efficiently block inflammation independent of GRα, PPARα, or PPARγ. Mol. Nutr. Food Res..

[6] De Keukeleire D. (2000). Fundamentals of beer and hop chemistry. Quím. Nova.

[7] Peng P., Wei W., Long C., Li J. (2017). Atorvastatin augments temozolomide’s efficacy in glioblastoma via prenylation-dependent inhibition of Ras signaling. Biochem. Biophys. Res. Commun..

[8] Sass G. (2012). Selective induction of apoptosis by HMG-CoA reductase inhibitors in hepatoma cells and dependence on p53 expression. Oncol. Rep..

[9] Zhang W., Li H., Yang Y., Liao J., Yang G.-Y. (2014). Knockdown or inhibition of aldo-keto reductase 1B10 inhibits pancreatic carcinoma growth via modulating Kras–E-cadherin pathway. Cancer Lett..

[10] Casey P.J. (1994). Lipid modifications of G proteins. Curr. Opin. Cell Biol..

[11] Chung Y.T., Matkowskyj K.A., Li H., Bai H., Zhang W., Tsao M.-S., Liao J., Yang G.-Y. (2012). Overexpression and oncogenic function of aldo-keto reductase family 1B10 (AKR1B10) in pancreatic carcinoma. Mod. Pathol..

[12] Endo S., Matsunaga T., Ohta C., Soda M., Kanamori A., Kitade Y., Ohno S., Tajima K., El-Kabbani O., Hara A. (2011). Roles of rat and human aldo–keto reductases in metabolism of farnesol and geranylgeraniol. Chem. Biol. Interact..

[13] Endo S., Matsunaga T., Mamiya H., Ohta C., Soda M., Kitade Y., Tajima K., Zhao H.-T., El-Kabbani O., Hara A. (2009). Kinetic studies of AKR1B10, human aldose reductase-like protein: Endogenous substrates and inhibition by steroids. Arch. Biochem. Biophys..

[14] Huang L., He R., Luo W., Zhu Y.-S., Li J., Tan T., Zhang X., Hu Z., Luo D. (2016). Aldo-Keto Reductase Family 1 Member B10 Inhibitors: Potential Drugs for Cancer Treatment. Recent Patents Anticancer Drug Discov..

[15] Cousido-Siah A., Ruiz F.X., Crespo I., Porté S., Mitschler A., Parés X., Podjarny A., Farrés J. (2015). Structural analysis of sulindac as an inhibitor of aldose reductase and AKR1B10. Chem. Biol. Interact..

[16] Soda M., Endo S., Matsunaga T., Zhao H.-T., El-Kabbani O., Iinuma M., Yamamura K., Hara A. (2012). Inhibition of human aldose reductase-like protein (AKR1B10) by α- and γ-mangostins, major components of pericarps of mangosteen. Biol. Pharm. Bull..

[17] Gallego O., Ruiz F.X., Ardevol A., Dominguez M., Alvarez R., de Lera A.R., Rovira C., Farres J., Fita I., Pares X. (2007). Structural basis for the high all-trans-retinaldehyde reductase activity of the tumor marker AKR1B10. Proc. Natl. Acad. Sci. USA.

[18] Cao D., Fan S.T., Chung S.S. (1998). Identification and characterization of a novel human aldose reductase-like gene. J. Biol. Chem..

[19] Martin H.-J., Breyer-Pfaff U., Wsol V., Venz S., Maser E. (2005). Purification and characterization of AKR1B10 from human liver: Role in carbonyl reduction of xenobiotics. Drug Metab. Dispos..

[20] Yan R., Zu X., Ma J., Liu Z., Adeyanju M., Cao D. (2007). Aldo–keto reductase family 1 B10 gene silencing results in growth inhibition of colorectal cancer cells: Implication for cancer intervention. Int. J. Cancer.

[21] Matkowskyj K.A., Bai H., Liao J., Zhang W., Li H., Rao S., Omary R., Yang G.-Y. (2014). Aldoketoreductase family 1B10 (AKR1B10) as a biomarker to distinguish hepatocellular carcinoma from benign liver lesions. Hum. Pathol..

[22] Quinn A.M., Harvey R.G., Penning T.M. (2008). Oxidation of PAH *trans* -Dihydrodiols by Human Aldo-Keto Reductase AKR1B10. Chem. Res. Toxicol..

[23] Ruiz F.X., Gallego O., Ardèvol A., Moro A., Domínguez M., Alvarez S., Alvarez R., de Lera A.R., Rovira C., Fita I. (2009). Aldo-keto reductases from the AKR1B subfamily: Retinoid specificity and control of cellular retinoic acid levels. Chem. Biol. Interact..

[24] Wang R., Wang G., Ricard M.J., Ferris B., Strulovici-Barel Y., Salit J., Hackett N.R., Gudas L.J., Crystal R.G. (2010). Smoking-Induced Upregulation of AKR1B10 Expression in the Airway Epithelium of Healthy Individuals. Chest.

[25] Reddy K.A., Kumar P.U., Srinivasulu M., Triveni B., Sharada K., Ismail A., Reddy G.B. (2017). Overexpression and enhanced specific activity of aldoketo reductases (AKR1B1 & AKR1B10) in human breast cancers. Breast.

[26] O’connor T., Ireland L.S., Harrison D.J., Hayes J.D. (1999). Major differences exist in the function and tissue-specific expression of human aflatoxin B1 aldehyde reductase and the principal human aldo-keto reductase AKR1 family members. Biochem. J..

[27] Barski O.A., Tipparaju S.M., Bhatnagar A. (2008). The Aldo-Keto Reductase Superfamily and its Role in Drug Metabolism and Detoxification. Drug Metab. Rev..

[28] Van Cleemput M., Cattoor K., De Bosscher K., Haegeman G., De Keukeleire D., Heyerick A. (2009). Hop (Humulus lupulus)-derived bitter acids as multipotent bioactive compounds. J. Nat. Prod..

[29] Shindo S., Tomatsu M., Nakda T., Shibamoto N., Tachibana T., Mori K. (2002). Inhibition of Aldose Reductase Activity by Extracts from Hops. J. Inst. Brew..

[30] Seliger J.M., Misuri L., Maser E., Hintzpeter J. (2018). The hop-derived compounds xanthohumol, isoxanthohumol and 8-prenylnaringenin are tight-binding inhibitors of human aldo-keto reductases 1B1 and 1B10. J. Enzyme Inhib. Med. Chem..

[31] Yamamoto K., Wang J., Yamamoto S., Tobe H. (2000). Suppression of cyclooxygenase-2 gene transcription by humulon of beer hop extract studied with reference to glucocorticoid. FEBS Lett..

[32] Hara A., Endo S., Matsunaga T., Soda M., El-Kabbani O., Yashiro K. (2016). Inhibition of aldo-keto reductase family 1 member B10 by unsaturated fatty acids. Arch. Biochem. Biophys..

[33] Zhang L., Zhang H., Zhao Y., Li Z., Chen S., Zhai J., Chen Y., Xie W., Wang Z., Li Q. (2013). Inhibitor selectivity between aldo-keto reductase superfamily members AKR1B10 and AKR1B1: Role of Trp112 (Trp111). FEBS Lett..

[34] Copeland R.A. (2004). Enzymes: A Practical Introduction to Structure, Mechanism, and Data Analysis.

[35] Dixon M. (1972). The graphical determination of Km and Ki. Biochem. J..

[36] Cattoor K., Bracke M., Deforce D., De Keukeleire D., Heyerick A. (2010). Transport of Hop Bitter Acids across Intestinal Caco-2 Cell Monolayers. J. Agric. Food Chem..

[37] Cattoor K., Remon J.-P., Boussery K., Van Bocxlaer J., Bracke M., De Keukeleire D., Deforce D., Heyerick A. (2011). Bioavailability of hop-derived iso-α-acids and reduced derivatives. Food Funct..

[38] Mukai R. (2018). Prenylation enhances the biological activity of dietary flavonoids by altering their bioavailability. Biosci. Biotechnol. Biochem..

[39] Karabín M., Hudcová T., Jelínek L., Dostálek P. (2016). Biologically active compounds from hops and prospects for their use. Compr. Rev. Food Sci. Food Saf..

[40] De Smet P.A.G.M., Keller K., Hänsel R., Chandler R.F. (1997). Adverse Effects of Herbal Drugs.

[41] Kao T.H., Wu G.Y. (2013). Simultaneous determination of prenylflavonoid and hop bitter acid in beer lee by HPLC-DAD-MS. Food Chem..

[42] Skarydova L., Tomanova R., Havlikova L., Stambergova H., Solich P., Wsol V. (2014). Deeper insight into the reducing biotransformation of bupropion in the human liver. Drug Metab. Pharmacokinet..

[43] Copeland R.A. (2000). Enzymes: A Practical Introduction to Structure, Mechanism, and Data Analysis.

[44] Bisswanger H. (2008). Enzyme Kinetics.

